# Two-photon calcium imaging during fictive navigation in virtual environments

**DOI:** 10.3389/fncir.2013.00104

**Published:** 2013-06-06

**Authors:** Misha B. Ahrens, Kuo Hua Huang, Sujatha Narayan, Brett D. Mensh, Florian Engert

**Affiliations:** ^1^Department of Molecular and Cellular Biology, Harvard UniversityCambridge, MA, USA; ^2^Janelia Farm Research Campus, Howard Hughes Medical InstituteAshburn, VA, USA

**Keywords:** zebrafish, virtual reality, two-photon calcium imaging, behavior, sensorimotor transformations, motor control

## Abstract

A full understanding of nervous system function requires recording from large populations of neurons during naturalistic behaviors. Here we enable paralyzed larval zebrafish to fictively navigate two-dimensional virtual environments while we record optically from many neurons with two-photon imaging. Electrical recordings from motor nerves in the tail are decoded into intended forward swims and turns, which are used to update a virtual environment displayed underneath the fish. Several behavioral features—such as turning responses to whole-field motion and dark avoidance—are well-replicated in this virtual setting. We readily observed neuronal populations in the hindbrain with laterally selective responses that correlated with right or left optomotor behavior. We also observed neurons in the habenula, pallium, and midbrain with response properties specific to environmental features. Beyond single-cell correlations, the classification of network activity in such virtual settings promises to reveal principles of brainwide neural dynamics during behavior.

## Introduction

Recording neuronal activity during behavior has a rich history. Early studies in freely moving animals involved extracellular recordings in rats (O'Keefe and Dostrovsky, 1971), and progressed to multi-unit electrical recordings (Churchland et al., 2012), and whole-cell (Lee et al., 2009), optical (Flusberg et al., 2008) and wireless recordings (Szuts et al., 2011; Yartsev et al., 2011). However, there are important limitations to approaches that require implanting recording devices in the heads of behaving animals. Most critically, the devices have to be small and this presents serious challenges for many advanced techniques such as two-photon microscopy and targeted whole-cell recordings. Consequently, efforts have intensified to develop virtual environments for animals, building on the long tradition of tethering insects for more controlled stimulus delivery (Gotz, 1968). The first such virtual-reality setup was used in mice to test multiple theories of place-cell mechanisms (Harvey et al., 2009; Dombeck et al., 2010), and to study mechanisms of decision making (Harvey et al., 2012). Similar setups for flying (Maimon et al., 2010) and walking (Seelig et al., 2010) fruit flies enabled optical and whole-cell recording of neurons in the visual system and other brain areas. Recently, a system was established for brain-wide optical recordings of neuronal activity during behavior in paralyzed larval zebrafish (Ahrens et al., 2012). In this work, however, fish were only able to control a single variable, namely the speed of a grating displayed at the bottom of the dish. This critically limited the types of behaviors that can be studied and thus begged the question whether such a set-up can be extended to navigation in two dimensions and, importantly, whether larval zebrafish will behave appropriately when immersed in such a highly artificial setting.

We therefore sought to develop a paradigm that would enable paralyzed fish to navigate through arbitrary visual environments. Paralysis in this context is not only advantageous for eliminating motion artifacts during imaging, it also abolishes all vestibular, proprioceptive and mechanosensory neural feedback that a behaving animal—be it tethered or untethered—usually receives. Uncontrolled neural feedback adds additional unknown variables which often interfere with the interpretation of recorded neural signals. In the paradigm described here, the experimenter is in the exceptional position of having full control of all sensory input to the animal. To establish a fictive two-dimensional paradigm, we first inspected the lateralized temporal patterns of motor nerve activity on the left and right sides of the tail during visually evoked swim events. We show that these signals contain sufficient information for decoding two-dimensional intended locomotion, and that they can be used to construct a two-dimensional closed-loop swim simulator for paralyzed zebrafish. Thus, we succeeded in translating the recorded motor signals into the fish's intent to turn and swim. Two independent tests were carried out to confirm the match between free swimming and fictive navigation. First, we examined the two-dimensional optomotor response (OMR) in both freely swimming and fictively navigating animals and found close matches in the behavioral statistics. Second, we found that fictively navigating fish reliably avoid dark areas in the virtual arena and that the detailed characteristics of this behavior closely resemble the statistics of freely swimming fish traversing a similar environment (Burgess and Granato, 2007). These results establish the possibility of investigating a range of behaviors in virtual environments, but also highlight some important differences between free and fictive behavior.

## Results

### Fictive motor patterns during directional visual stimulation

**Figure 1:**
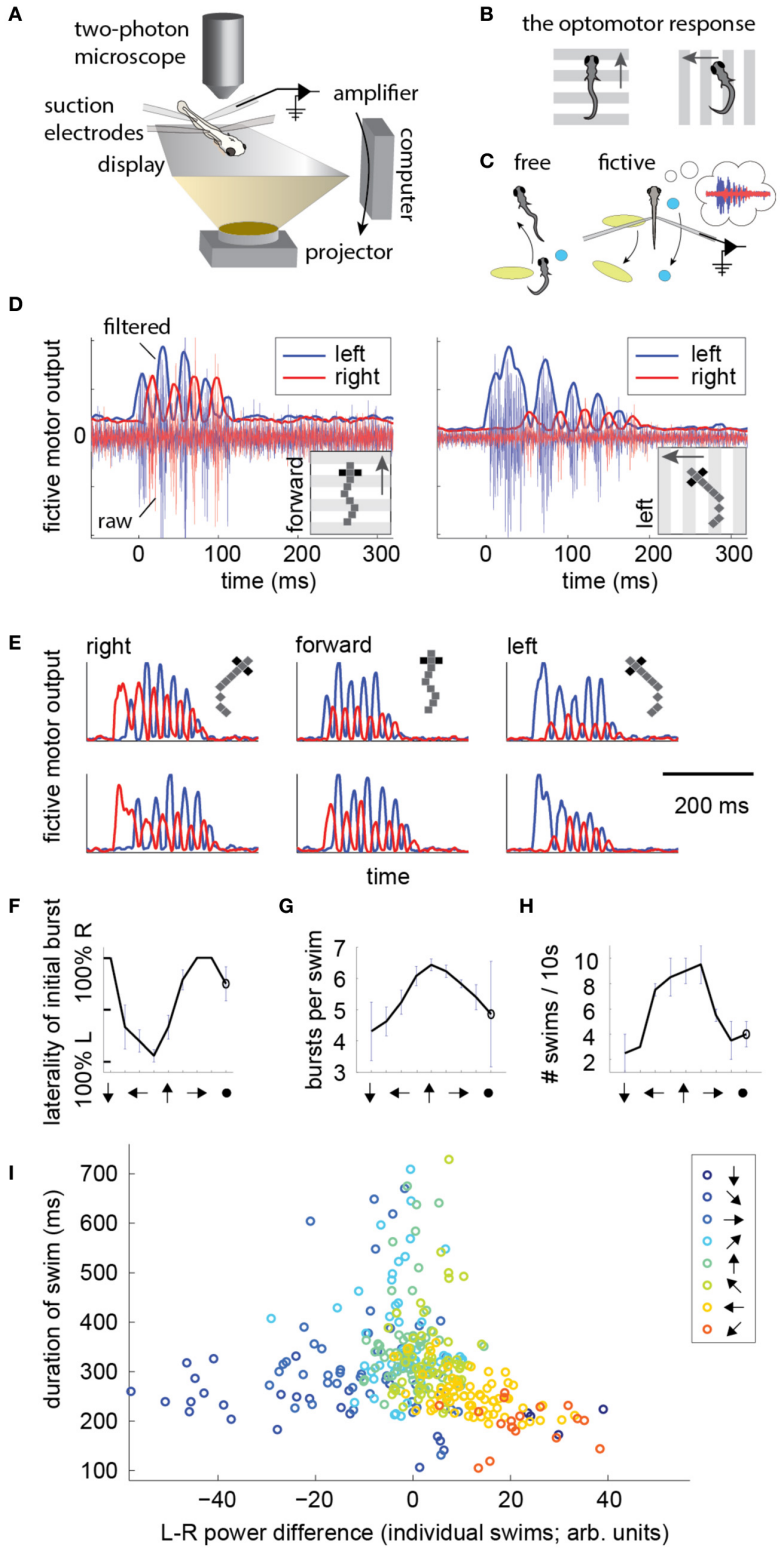
**Electrical signatures of fictive forward swims and turns in paralyzed fish. (A)** Fictive virtual-reality setup. A larval zebrafish is paralyzed, head-embedded in agar or suspended from pipettes, and suction electrodes record activity from motor neuron axons. Motor neuron activity is used to simulate movement through a virtual environment which is projected on a display underneath the fish. A two-photon microscope records neuronal activity as reported by a genetically-encoded calcium indicator. *Right:* schematic of the correspondence between free swimming behavior and fictive behavior. In each case, the movement of the world, as seen from the fish's point of view, is the same. **(B)** Schematic of the optomotor response (OMR) in a freely swimming fish (top view). Fish turn and swim along with the direction of whole-field motion.** (C)** Schematic of the goal of the current work. *Left:* a freely swimming fish makes a left turn (top view). *Right:* a fictively swimming paralyzed fish intends to make a left turn, upon which the virtual environment projected underneath the fish shifts to induce the same optical translation as observed by the freely swimming fish. **(D)** *Left:* example of a fictive forward swim. This was elicited by forward, tail-to-head whole-field motion. The pixelized fish indicates that this is fictive behavior. *Right:* example of a fictive turn to the left, elicited by leftward whole-field motion. Thin lines depict electrical signal, thick lines represent the local standard deviation in a running window of 10 ms. Data in panels **(D–H)** is from one representative fish. **(E)** Additional examples of fictive turns and forward swim bouts. **(F–H)** Statistics of responses to whole-field motion in a typical fish. Each point on the graph represents a different direction of whole-field motion. The last point represents a static scene. **(F)** Probability of above-threshold activity occurring on the left or the right electrode for a typical fish. This open-loop behavior is similar to “virtual open loop” behavior observed in freely swimming fish (Orger et al., 2000). **(G)** Number of bursts per swim is largest for forward visual stimulation. **(H)** Number of swim bouts in a 10 s window increases with larger forward component of visual motion. **(I)** Difference in power of the left and right channels as a function of the direction of visual stimulation, showing a systematic relationship between these two variables, suggesting that left-right power difference may code for intended turn angle.

To record intended locomotion in immobilized animals, larval zebrafish were paralyzed by immersion in a solution containing bungarotoxin, and either head-embedded in agarose, or suspended in mid-water from three suction pipettes (Methods). Next, two or four suction electrodes were attached to the tail at intersegmental boundaries to record multi-unit activity from motor neuron axons (Figure 1A) (Masino and Fetcho, 2005; Ahrens et al., 2012). (For four electrodes, on each side of the tail, the electrode with the higher signal to noise ratio was selected and the other was ignored). Visual stimulation was delivered using a display underneath the fish. To examine the temporal patterns of motor nerve activity during intended locomotion, we made use of a robust reflexive behavior known as the OMR (Orger et al., 2008). In the OMR, animals respond to whole-field visual motion by turning and swimming along with the direction of the moving stimulus (Figure 1B), which is thought to represent the fish's attempt to maintain a constant position relative to the ground in the presence of a water current. By presenting forward moving gratings, which normally evoke forward swimming, it became apparent that the electrical signature of forward swim bouts consists of symmetrically alternating activity on the left and right sides of the tail at a similar period as that of tail oscillations in freely swimming fish (Figure 1D, left; Figure 1E; pixelized fish indicates fictively behaving animal). Conversely, by presenting left-moving gratings, we observed high-amplitude, long-duration bursts of activity on the left electrode at the start of most swim bouts (Figure 1D, right; Figure 1E), which we interpret as representing the relatively stronger tail bend that initiates a turning behavior (Budick and O'Malley, 2000). These patterns were consistently present during presentation of whole-field motion in the forward, left, and right directions (Figure 1E). Further, after examining eight angles of whole-field motion, we found that statistics of these electrical signals (Figures 1F–H) qualitatively matched behavioral features of freely swimming animals responding to whole-field motion (Orger et al., 2008). This qualitative correspondence encouraged us to attempt to translate the electrical signals into a measure of motor intent (direction of swim), and to use this inferred intent to control its movement in a virtual environment.

**Figure 2:**
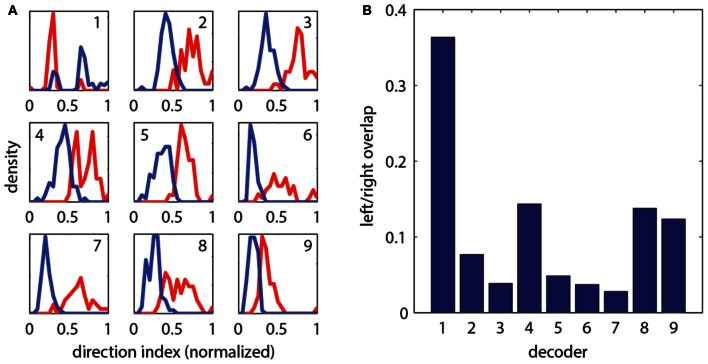
**Evaluation of different strategies for decoding fictive turn direction. (A)** Histograms of normalized direction index of nine different decoders, over 127 total swim bouts (one representative fish). Left and right moving gratings were presented to paralyzed fish while their fictive responses were recorded. Nine different decoders each produced different sets of direction indices (*red*: responses to right, *blue*: responses to left). Decoding strategies were (“bumps” refers to the oscillations visible in the fictive swim bouts, as in Figure 1): (1) Ratio of width of first left and right bumps of the processed fictive signal, (2) Ratio of heights of the first bumps, (3) Ratio of the left and right power of the entire swim bout, (4) Ratio of the areas of the first bump of the left and right swim bout, (5) Difference in the widths of the first bumps, (6) Difference in the heights of the first bumps, (7) Difference in left and right power of the entire swim bout, (8) Difference in the areas of the first bumps, (9) Difference in the rise time (time to peak) of the first bumps. It can be seen that each decoder produces segregation between the responses to the left and right moving visual stimuli, but some cause more segregation than others. **(B)** Quantification of overlap in direction index of the nine decoders (lower value indicates better performance). Decoder (7), the difference in power between the left and right channels over the entire swim bout, performs the best.

To construct a measure of intended swimming and turning, we hypothesized that the difference in power between the left and the right recording channels would provide information about the direction of intended locomotion. Figure 1I shows the difference in power among swims recorded during presentation of whole-field motion in eight different directions. A systematic shift in the power difference is observed when the angle of stimulation changes, peaking during backward-left and backward-right motion, consistent with freely swimming behavior (Orger et al., 2008). During forward motion, the left and right power equilibrates, as is expected from the symmetric undulations of the tail during forward swimming. We also surveyed eight other decoding strategies based on various features of fictive swim bouts (Figure 2). We found that the decoder that was based on the difference in power performed the best. Moreover, this decoder can compute angle change in real time by incrementally increasing or decreasing the heading angle according to the difference in power during each video frame. In contrast, decoders based on parameters such as the duration of the first oscillation of the swim bout only produce an angle change after the first oscillation has terminated. Thus, we constructed a swim decoder where the turning angle was taken to be proportional to a weighted difference in power on the left and right side of the tail and the strength of the forward swim was taken to be proportional to the sum of the power on both sides of the tail (Methods). We next tested whether this decoder is sufficient for driving closed-loop behavior in virtual environments.

### The two-dimensional optomotor response, real and fictive

The above motor patterns were measured in open-loop, where a fixed stimulus is presented and the fictive behavior monitored. Next we investigated if these results can be used to simulate two-dimensional swimming. To this end, we established a closed-loop swim simulator (Figure 1C), using the above measures as a readout of intended turning and forward swimming. In short, activity on the left side of the tail drove left rotation of the fish in a virtual environment (i.e., right rotation of the visual environment), activity on the right side drove right rotation of the fish (left rotation of the visual environment), and activity on both sides drove virtual forward swimming (backward movement of the visual environment). After tuning the three parameters of the decoder (Methods), i.e., the constants of proportionality for left turns, forward swims, and right turns, the OMR was evoked in a virtual environment and the behavior of the fish compared to the analogous free swimming behavior.

**Figure 3:**
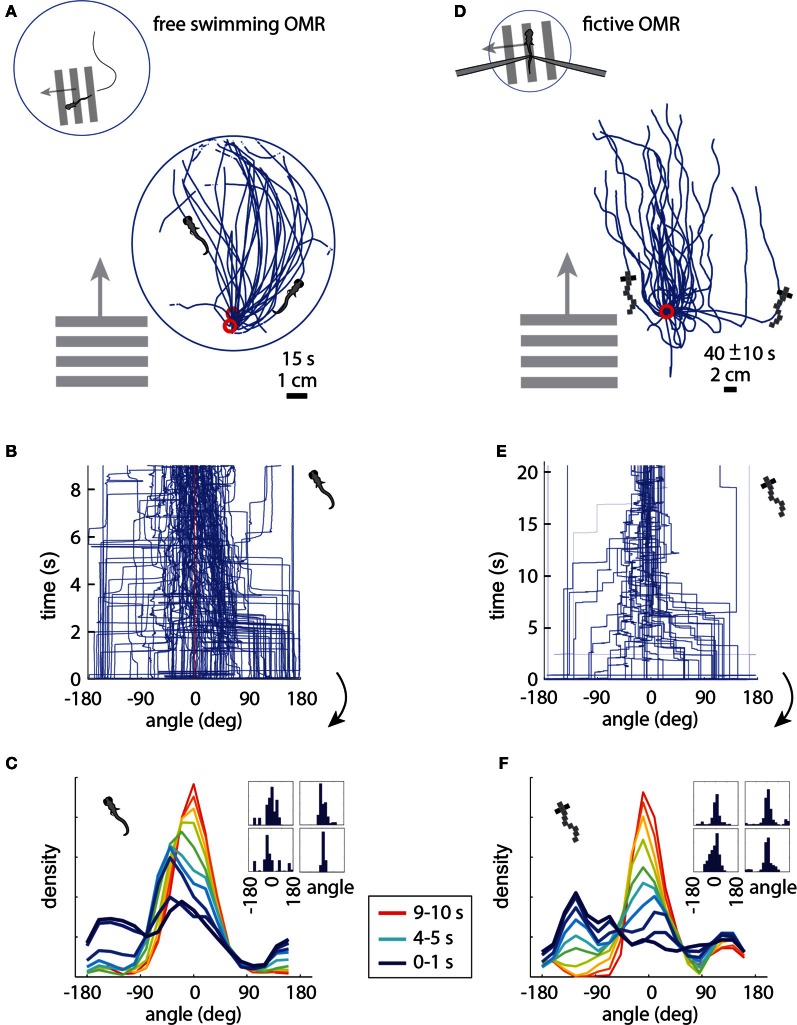
**The optomotor response in free (A–C) and paralyzed (D–F) fish. (A)** Swim trajectories during visual whole-field motion projected at the bottom of a 10 cm petri dish (*N* = 3 example fish). Grating period was 1 cm, speed 1 cm/s. Data have been rotated so that the starting point is near the bottom of the dish, as seen from above. **(B)** Heading angle over time for *N* = 6 freely swimming fish. The time axis is cut off at 9 s, because at that time a number of fish reached the end of the petri dish and the heading angle became ill-defined. **(C)** Changing heading direction over time; same data as **(B)**. *Dark blue:* histogram at start of trials (*N* = 6 fish, 10 trials each). *Red:* histogram 10 s after trial onset. Each color represents one second of data. *Inset:* Histograms of heading angle, centered at zero degrees, which is the direction of motion (4 representative fish). **(D)** Fictive swim trajectories in the virtual reality version of the OMR. Like freely swimming fish, the paralyzed fish turn in the direction of motion and swim along with it (*N* = 6 fish). **(E)** Heading direction over time for *N* = 6 paralyzed fish. Note that the time axis is different from **(B)** (because distance in the virtual environment was not limited by a petri dish), but the dynamics are similar (see panels **C** and **F**). **(F)** Changing heading direction over time (*N* = 6 fish; same data as **E**). Colors as in **(C)**. The time course of heading angle change is similar to the freely swimming case shown in **C**. *Inset:* Angle histograms of four representative fish over the entire experiment.

In the free swimming OMR, a whole-field visual stimulus, in this case black and white bars, is moved at a fixed velocity in a fixed direction, which causes the zebrafish to turn and swim along the direction of motion (Video S1). Figure 3A shows the behavior of three fish performing the OMR. The fish swam around in a 10 cm diameter petri dish that was positioned above a display screen (Methods). At a time point when the fish was close to the edge of the dish (but not touching), whole-field motion was initiated in the direction perpendicular to the wall at the location of the fish. In Figure 3A, the paths traced out by the fish are rotated so that the starting position is always at the bottom of the petri dish and the direction of the motion stimulus is upward. Consistent with previous reports of the OMR (Orger et al., 2008), the fish turned and swam in the direction of motion, with the angle between the heading direction and the direction of motion over all 15 s trials centered around 0° (Figures 3B,C). At initiation of stimulus movement, the fish faced a random direction (Figure 3C, dark blue). They then turned to adjust their heading direction toward that of the motion (Figure 3C, blue-red gradient), and this process equilibrated after about 5–10 s (Figure 3C, yellow-red). With this characterization of the free swimming OMR, we now turned to the analysis of the fictive OMR in the virtual environment.

In the virtual reality OMR paradigm (see Video S2 for a more intuitive demonstration of the closed-loop dynamics), the fish was similarly faced with whole-field visual motion, and the virtual fish was periodically “spun round” to randomize the heading direction. The direction of visual motion is then controlled in closed loop by the fish's fictive swims (Figure 1C). For example, if the direction of optic flow is to the left and the fish makes a fictive turn to the left, we rotate the visual environment to the right, resulting in the new direction of optic flow being forward (tail to head). This new optic flow can be slowed down or even accelerated backward by a fictive forward swim, simulating forward swimming. Tracing the virtual swim paths in the simulated environment, a similar pattern appears as in freely swimming fish: paralyzed fish efficiently turn and swim along with the direction of optic flow (compare Figure 3D to the free swimming case in Figure 3A).

We measured several parameters of the OMR in the virtual environment and found that the fictive swimming behavior of paralyzed zebrafish is strikingly similar to that of freely swimming fish. The heading angle is clustered around the angle of visual motion, as in the freely swimming case, and to a similar extent (compare the fictive case in Figures 3E,F to the free case in Figures 3B,C). When the direction of motion in the virtual environment is changed to a random angle, the fictively swimming fish corrects for this over the course of 5–10 s, as is also observed in the freely swimming fish (compare Figure 3F to Figure 3C).

Given the open-loop results (Figure 1), it is not surprising that the paralyzed fish intend to turn and swim in the expected direction. It is, however, remarkable that these neural measures of motor output can be used in a straightforward manner to simulate a two-dimensional assay, such that the fish displays naturalistic behavior, with multiple measures closely matched between free and paralyzed animals.

### Imaging during the optomotor response

**Figure 4:**
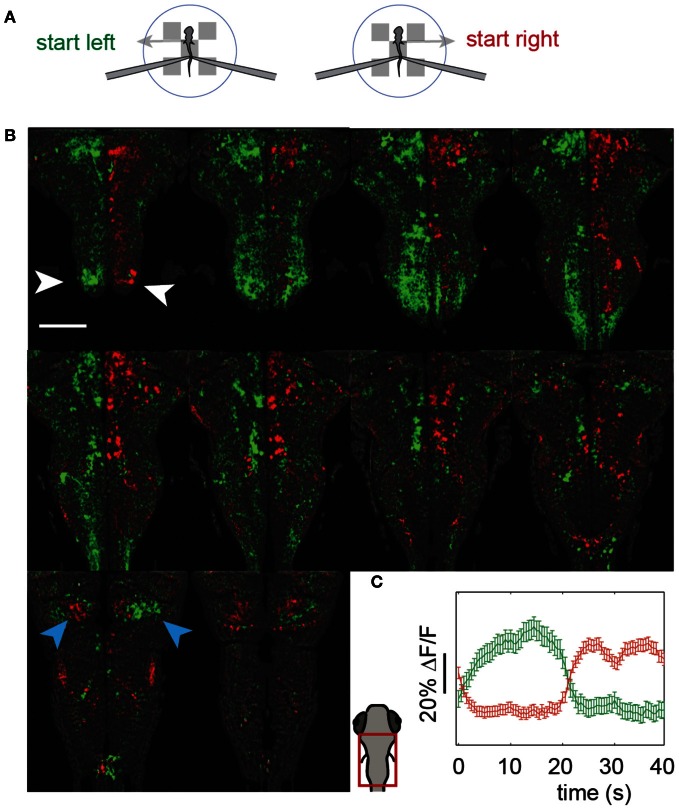
**Whole-hindbrain imaging during left/right biased optomotor behavior. (A)** Summary of fictive behavioral assay. The assay was identical to the optomotor assay (Figure 3), but the angle was perturbed every 10 s and set to leftward (0 s), leftward (10 s), rightward (20 s), rightward (30 s), repeating. In this way, the fish was biased to turn left during 20 s, and to turn right during 20 s. This sequence was repeated twice, over a period of 80 s, for each imaged plane. **(B)** Two-photon imaging of most of the hindbrain during the assay. The imaging area is shown in the schematic of the larval head. Planes 5 μm apart were imaged each for 80 s **(A)**. Data are displayed as the average of three consecutive planes (i.e., 15 μm between panels), and represent the difference in average calcium signal over the left (*green*) and the right (*red*) periods (i.e., activity during 0–20 and 40–60 s, minus activity during 20–40 and 60–80 s, for each plane); for the ΔF/F scale over time, see **(C)**. An approximate lateral organization of activity during the left and right periods can be seen, including in the inferior olive (*white arrows*). The polarization is flipped in regions of the cerebellar cortex (*blue arrows*), consistent with cerebellar anatomy; climbing fibers from the inferior olive cross contralaterally before projecting to the cerebellar cortex. Scale bar, 100 μm. **(C)** Average activity of the red and green populations over the 40 s of the assay **(A)**. Error bars: standard error across neurons (including neuron-sized areas of neuropil).

We imaged the entire hindbrain while zebrafish performed the two-dimensional OMR, in search of neural correlates of left and right optomotor behavior, in a transgenic fish, Tg(*elavl3:*GCaMP5) (Ahrens et al., 2013), expressing the genetically encoded calcium indicator GCaMP5 (Akerboom et al., 2012) under the *elavl3* promoter (Park et al., 2000) in almost all neurons. To this end, we initialized the direction of whole-field motion to 90° (left) and −90° (right) with respect to the fish's body (Figure 4A: left initializations at 0 s and 10 s, right at 20 s and 30 s, with two 40 s repetitions per imaged plane). Subsequently, the fish was able to control its movement in the virtual environment and swim along with the direction of motion. We were able to detect differences in neural activity during the left and right phases by reconstructing the entire hindbrain volumes from the individually imaged planes and subtracting the volumes corresponding to these two periods from one another. Figure 4B shows the hindbrain volume (each panel is the average of three consecutive 5 μm planes; see also Video S3). A left-right segregation of activity during left and right optomotor behavior is observed, including in the inferior olive (white arrows). In the cerebellar cortex, this lateralization partially reverses (blue arrows), probably because climbing fibers from the inferior olive cross to the contralateral side before projecting to the cerebellar cortex. Figure 4C shows activity of the two neural populations, averaged over the 40 s periods. Thus, activity in entire brain regions, and by extension in the entire brain, can be mapped with a two-photon microscope during fictive navigation.

**Figure 5:**
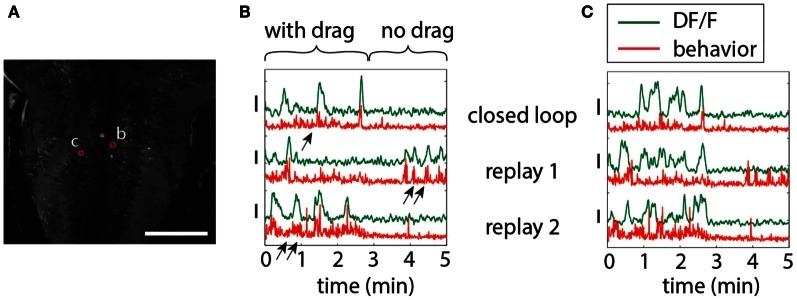
**Differences in neuronal responses to closed-loop and open-loop visual stimulation**. Five minutes of closed-loop virtual behavior was followed by two replay runs, during which the recorded visual stimulus was played back in open-loop. In closed-loop, a period with drag (forward stimulus motion in the absence of swimming, simulating a backward water current) was followed by a period without drag (no stimulus motion in the absence of swimming). **(A)** Hindbrain anatomy, with neurons b and c marked in red. Scale bar: 100 μm. **(B)** Motor-related neuronal activity, with increased fluorescence signal during more vigorous swimming. Note that the behavior, as well as the neuronal activity, is different during closed-loop and open-loop replay, underlining the importance of closed-loop virtual behavior above simple open-loop stimulus presentation. Behavior, as also shown before (Ahrens et al., 2012), is qualitatively different in closed- and open-loop, with open-loop behavior often more vigorous (double arrows) than closed-loop behavior (single arrow). **(C)** Non-motor-related neuron activity that may be visually driven. Patterns of activity during closed- and open-loop are similar, but not identical, and bear no correlation to motor output (e.g., see replay 1). This suggests that this cell is driven by visual input, albeit in a non-trivial way.

To illustrate the importance of environmental feedback, we compared behavior in open-loop and closed-loop conditions. A closed-loop period was followed by two repetitions of the stimulus in open loop, so that the visual patterns in both open and closed loop were identical, but in closed loop were self-generated. Figure 5 shows two neurons in the hindbrain, each exhibiting different temporal patterns of activity during closed-loop and open-loop replay. Activity of neuron **b** is determined by motor output; this neuron may drive behavior or receive feedback from motor centers. Importantly, behavior during closed- and open-loop is different (double arrows vs. single arrows; behavior during open loop tends to be more vigorous), so that the function of this neuron cannot be investigated solely by presenting visual stimuli in open-loop. On the other hand, neuron **c** appears to be driven by visual stimulation, with similar activity patterns during open-loop replay; however, differences still exist, suggesting that this neuron may be partially driven by behavior or other internal processes.

### Phototaxis in a virtual environment

**Figure 6:**
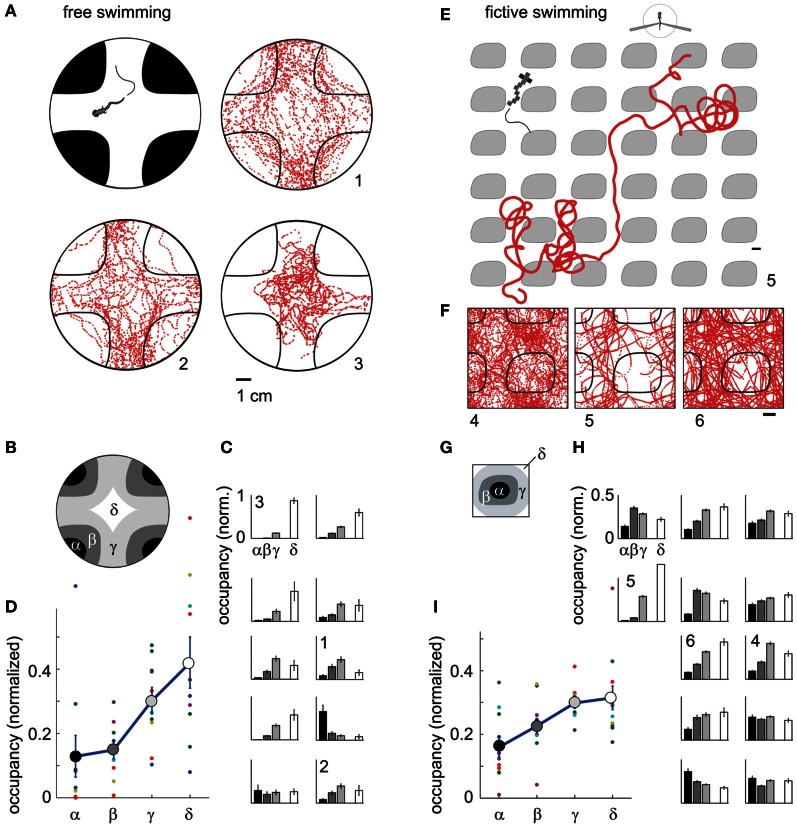
**Darkness avoidance in free and paralyzed fish. (A)** Top view schematic of the arena, consisting of a 10 cm petri dish on top of a visual display. Display color was red to replicate the color used during two-photon imaging. Shown are trajectories of 3 fish over 20 min of experimental time (labeled 1–3). On average, fish spend more time in the bright region. **(B)** Subdivisions α − δ of the arena used for further analysis of location preference. **(C)** Time spent in the areas α − δ (*N* = 10 fish). Eight fish preferentially stay in the bright region; one fish spends more time in the dark region; one fish does not distinguish. Error bars computed from Bernoulli distribution over counts, then normalized to the area of the four regions. **(D)** Average normalized occupancy of regions α − δ (*N* = 10 fish) shows a strong and graded bias toward staying in the bright regions. **(E)** Example trajectory of paralyzed fish through the virtual environment. Scale bar, 1 cm. **(F)** Complete trajectories of three fish over all tiles of the virtual environment, shown in relation to a single tile and parts of neighboring tiles. **(G)** Subdivisions of one tile, with α and β the center and boundary of a dark tile, and δ and δ inner and outer areas of the bright space, as in **(B)**. **(H)** Occupancy over areas α − δ of all tiles in the virtual environment (*N* = 12 paralyzed fish). Most fish spend less time in the dark. Some also avoid the center of the white areas (δ), such as fish 4. **(I)** Average normalized occupancy of bright to dark regions α − δ of the virtual environment shows graded preference to bright areas. Differences in occupancy are smaller than in freely swimming fish **(D)**.

Next, we used phototaxis as a second behavior to test the capabilities of the fictive-virtual paradigm for mimicking natural behavior. Larval zebrafish are attracted to light and avoid darkness (Burgess and Granato, 2007), a behavior that may allow them to avoid deep water, the shadows of predators, and dimly lit areas where it may be hard to detect both predators and food. To quantify phototaxis in freely swimming fish we observed their swimming behavior in an environment consisting of bright and dark areas (Figure 6A), (Methods). We found that animals spent significantly more time in the bright areas than in the dark areas (Figures 6A–D; *p* < 0.001, *t*-test, *N* = 10 fish), although variations existed across the 10 fish tested (Figure 6C).

Transitioning to the fictive virtual-reality paradigm, we implemented a similar environment, consisting of infinitely repeating black tiles on a white background, through which the fish could fictively swim. Unlike in the fictive OMR paradigm, there was no constant flow present, so that the visual environment remained stationary in the absence of fictive locomotion. In this virtual environment, most fish avoided the dark areas (Figures 6E–I, Video S4), preferring to stay in the bright regions (*p* < 0.001, *t*-test, *N* = 12 fish).

Some freely swimming fish avoided the bright inner region of the arena in addition to the dark region (e.g., Figures 6A and C, fish 1,2). Similar behavior was present in some fictively navigating fish (e.g., Figures 6F and H, fish 4). Overall, the degree of darkness avoidance was stronger in freely swimming fish than in fictively behaving fish, as can be seen from the comparison of the normalized occupancy of the dark and bright regions show in Figures 6D,I. This demonstrates that not all quantifiable elements of a behavior are guaranteed to equate between the freely swimming to the fictive case, even if the core features of the behavior are qualitatively preserved.

In summary, we have shown that paralyzed larval zebrafish can traverse a light-modulated environment and show similar attraction and repulsion behavior as freely swimming fish. Combined with the results from the optomotor behavior, this indicates that fictive navigation robustly recapitulates natural behavior.

### Functional imaging during fictive-virtual navigation

We have shown that paralyzed zebrafish are able to meaningfully traverse two dimensional environments. We asked whether the activity of neurons in the forebrain carry representations of features of the environment, and whether these can be detected by two-photon calcium imaging while the animals are navigating. In the light-modulated virtual environment, we sought neurons that responded to bright and dark features, with a particular focus on the habenula and pallium (Northcutt et al., 2004; Zhao and Rusak, 2005), and found activity that was both positively and negatively modulated by light level in both regions.

**Figure 7:**
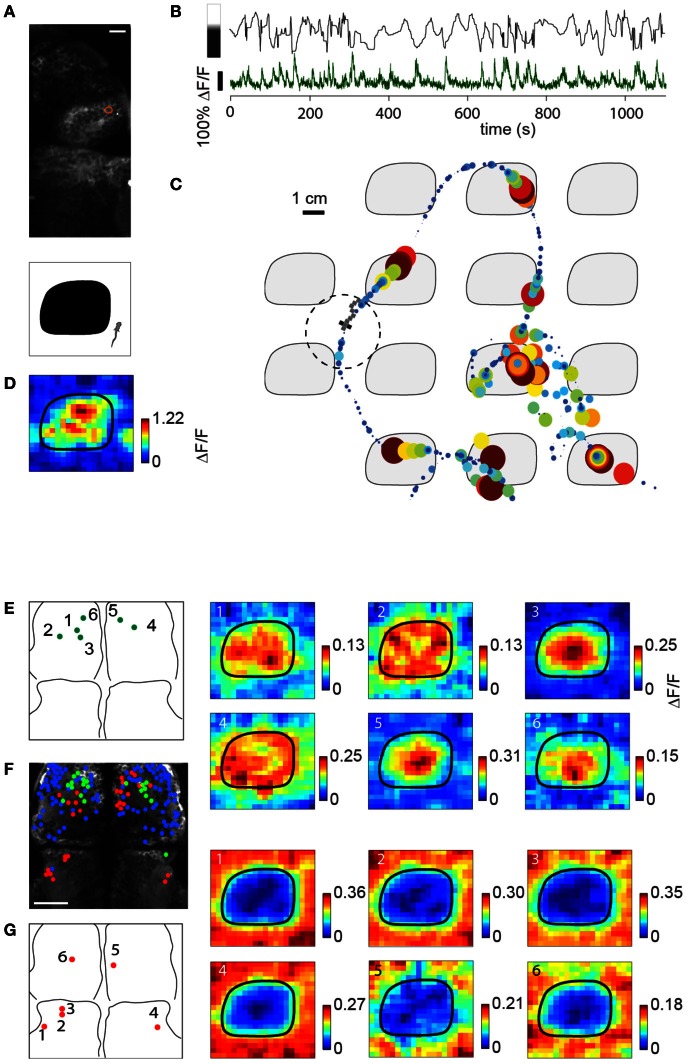
**Imaging sensory activity during fictive navigation. (A)** *Top:* Two-photon image of right anterior midbrain and forebrain, with neuron displayed in **(B–D)** circled in red. Scale bar: 20 um. *Bottom:* schematic of one tile of the virtual environment. **(B)** Activity of neuron outlined in **(A)** during fictive navigation of a transgenic *elavl3:GCaMP5* fish expressing a genetically-encoded calcium indicator in almost all neurons. *Top:* Distance from the center of the nearest dark patch, with bar on the left indicating approximate brightness. *Bottom:* ΔF/F. Peaks in ΔF/F coincide with approaches to the center of a dark patch. **(C)** Neural activity overlaid on the path through the virtual environment. Activity is represented by size and color, with largest discs corresponding to ΔF/F = 1. Seven minutes of the experiment are shown.** (D)** Neural activity averaged over the entire trajectory, averaged over one tile. This activity pattern is consistent with a neuron activated by darkness. **(E)** *Left:* Locations of six dark-responding neurons in the pallium of a fictively navigating fish with activity patterns shown on the right. *Right:* Activity of these neurons is similarly modulated as in **(D)** while this paralyzed fish traverses the light-modulated environment. Details of the activity maps vary; for example, high activity of neurons 5 and 6 is constrained to the center of the black patches, whereas that of neurons 2 and 4 is more broadly distributed within patches. **(F)** Anatomy of anterior midbrain and pallium of this fish, with neuron positions superimposed. *Green:* neurons responding to dark areas, *red:* neurons responding to bright areas, *blue:* neurons with no significant location-dependent change in activity. **(G)** *Left:* Locations of six light-responsive neurons (2,3,5,6) and neuropil regions (1,4), *right:* response maps of these neurons, showing suppression of activity when the fish is on a dark patch, and excitation when it is on a bright region.

Figure 7A shows the location of a neuron in the habenula of a Tg(*elavl3:*GCaMP5) fish traversing the light-modulated environment. The activity of this neuron fluctuates over time and is partially determined by whether the fish is on top of a dark patch or bright region (Figure 7B). Representing neuronal activity as a function of location in Figure 7C, elevations in the calcium readout can be observed as the fish enters a dark patch (7 minutes of data shown). Since the environment consists of repeating tiles, ΔF/F can be represented as an average over a single tile (Figure 7D).

Subpopulations of neurons in the pallium also had excitatory responses to darkness, which could be detected during virtual navigation (Figures 7E,F, green). Another population (Figure 7F, red; Figure 7G) responded to light and was suppressed by darkness (5,6 in pallium; 1–4 in anterior midbrain, 1 and 4 are neuropil, the rest are neurons). The fact that activity in these neurons—likely modulated by light level around the fish, but possibly also by other features (e.g., panel **E**, compare neurons 1,2,4 to neurons 3,5,6)—can be mapped to spatial location as the fish fictively navigates opens up the possibility to study more general representations of features of the environment across the entire brain.

**Figure 8:**
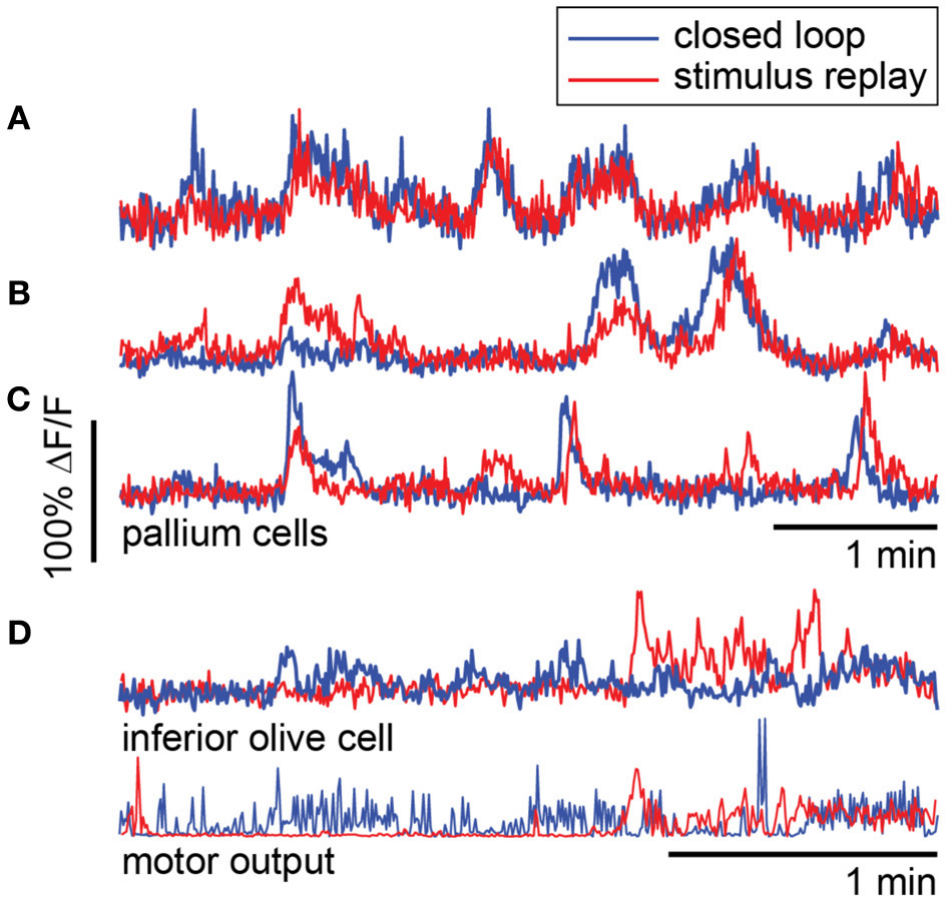
**Forebrain activity during closed- and open-loop navigation in a dark-modulated environment is largely stimulus-locked. (A–C)** Recordings from pallial neurons during two-dimensional darkness avoidance. For 5 min, fish navigated a virtual environment. Next, the stimulus presented during this period was repeated in open-loop. Neural activity during the first 5 min shown in blue; activity during the five minutes of stimulus replay shown in red. Neurons a–c are in the same fish, over the same period. **(A)** Activity in this neuron is identical during the closed- and open-loop periods, indicating that this is a visually-driven neuron with activity that is highly predictable from the visual stimulus alone. **(B,C)** Two neurons that are stimulus-driven, but whose activity is differently modulated during closed-loop and open-loop replay. Differences in activity may represent differences in the state of the brain during closed- and open-loop control. **(D)** For comparison, a neuron in the inferior olive with activity that is different during closed-loop and open-loop replay. Activity in this neuron correlates with motor output (bottom). Data were obtained using a one-dimensional OMR assay (Ahrens et al., 2012). Swim patterns during closed- and open-loop are qualitatively different, with behavior in closed-loop being more ongoing, and behavior in open-loop characterized by periods of quiescence and periods of vigorous swimming. These patterns are also reflected in the neural activity.

To compare closed- to open-loop conditions, we show in Figures 8A–C the activity of three neurons in the pallium during navigation through the light-modulated environment, and the subsequent stimulus-replay period. Neuron **a** is faithfully driven by the stimulus—its activity pattern during stimulus replay is identical to the activity during navigation. Neurons **b** and **c** are also sensory neurons, with similar activity during navigation and stimulus replay; however, during replay, activity is modulated differently, which may be due to a change in behavioral state, possibly induced by the open-loop condition. For comparison, neuron **d**, recorded in a different fish during an optomotor paradigm (Ahrens et al., 2012), exhibits very different activity during open-loop stimulus replay, evidenced by the change in behavioral patterns during closed- and open-loop (Figure 8D, bottom). Together with Figure 5, these results emphasize the fact that stimulus delivery alone is not sufficient for investigating neural activity during sensorimotor transformations.

## Discussion

Neural systems have evolved to generate behavior guided by sensory input. Although much can be learned from studying sensory (Hubel and Wiesel, 1959) and motor (McLean et al., 2008; Smetana et al., 2010) systems in isolation, it is becoming increasingly clear that a full understanding of neural circuits relies on observing and perturbing neuronal activity while the animal is actually behaving (Chiappe et al., 2010; Maimon et al., 2010). The larval zebrafish occupies a unique position in neuroscience because it is transparent and small, so that optical measurement of neuronal activity anywhere in the brain is possible (Ahrens et al., 2012). However, by the same token, it is too small to carry a mobile neural recording apparatus. This highlights the importance of the system we present here, as it allows us to measure and perturb neuronal activity at the single-cell level across the whole brain during fictive navigation. The demonstration that paralyzed larval zebrafish navigate similarly to freely swimming animals suggests that it is possible to use this system to study neural mechanisms underlying many behaviors, including associative learning (Aizenberg and Schuman, 2011), two-dimensional motor adaptation (Ahrens et al., 2012), and possibly place-preference learning (Ofstad et al., 2011). In addition, neural representations of spatial features or the location of the animal in space (Vargas et al., 2006) may exist in the brain of the larval zebrafish. It is now possible to search for these throughout the brain of the larval zebrafish without any *a priori* bias for specific brain regions or nuclei.

An aequorin based method for monitoring neural activity in behaving zebrafish was reported previously (Naumann et al., 2010). In this study, a bioluminescence system reports calcium influx by emitting photons without the need for the delivery of excitation light, and thus allows for non-invasive recording of activity in freely swimming animals. However, this system has no spatial resolution, and makes it therefore difficult to assign measured signals to multiple unique neurons. Instead, neuronal specificity has to be achieved by genetically restricted expression (Scott et al., 2007) of the protein. The aequorin system and fictive virtual reality are complementary methods for monitoring neural activity in behaving zebrafish.

In the experiments described here, visual feedback was the only feature that informed the animal about locomotor events. Under normal conditions this gets supplemented by a variety of additional reafferent signals like proprioception, lateral line and vestibular input. As such, it is surprising that the paralyzed zebrafish behave so similarly to freely swimming animals. Although this does not imply that sensory feedback from other modalities is unimportant, it is consistent with the hypothesis that the nervous system is able to adapt to the feedback that is readily available and learns to ignore missing signals from other modalities. Furthermore, it is experimentally convenient, in particular because photons from the visual display do not move the sample. Importantly, the experimenter has full control over, and full knowledge of, all dynamic sensory input driving the nervous system of the fish, eliminating unknown variables such as responses of hair cell spike patterns to turbulent water. At the same time, it is possible to add further modalities, such as swim-triggered water flow that mimics somatosensory feedback during swim bouts, although it may be technically challenging to accurately mimic the hydrodynamics of freely swimming animals.

We did observe differences between free and fictive behavior, most notably in the specificity of darkness avoidance. This may arise from three sources. First, it is possible that the avoidance response is changed due to the paralysis of the zebrafish, raised stress levels, or lack of non-visual sensory feedback. Second, imperfections in the decoding of intended locomotion from electrical signals in the motor neuron axons may make it harder for the fish to navigate as accurately as it would if it were freely swimming. Third, α-bungarotoxin, which blocks the nicotinic acetylcholine receptor at the postsynaptic site of the neuromuscular junction, also blocks similar receptors in the brain, which may lead to changes in behavior—something that should be taken into account whenever drawing parallels between fictive and free behavior. Improvements in the decoding algorithm, and possibly an increase in the number of electrodes recording motor signals, may increase the accuracy of the decoded trajectory.

The hindbrain imaging results demonstrate the ease with which entire brain areas can be interrogated for representations of visuomotor actions: the activity maps were computed by simply subtracting two volumes, acquired at different phases, from one another. This analysis was sufficient for uncovering subtle phenomena such as the reversal of direction preference in the cerebellum compared to the inferior olive. More advanced analysis methods will lead to deeper insights into circuits involved in sensorimotor processing.

Our mapping of neuronal forebrain activity onto virtual space demonstrates that the fictive virtual reality paradigm can be used to investigate neural representations of features of the visual environment. Although it is possible that the observed responses contain place-cell like representations of higher-order features of the environment, the simplest explanation of their activity patterns is that they are light or dark sensitive. Visual responses have been observed in the habenula of rats (Zhao and Rusak, 2005) and in the pallium of bony fish (Northcutt et al., 2004), but to our knowledge have not yet been reported in these brain areas in larval zebrafish.

The virtual reality setup not only enables neuronal recordings during fictive behavior, but also electrophysiology and perturbation of neural activity via optogenetic tools (Fenno et al., 2011) and single-neuron ablation (Orger et al., 2008). Electrophysiology may be used to measure and perturb single-cell intracellular voltage at high temporal resolution; this would complement calcium measurements which have low temporal resolution and are not well suited for detecting hyperpolarizations. Optogenetic tools can be readily applied to test causal relationships between neural activity and behavior. Furthermore, the setup can, in principle, be combined with light-sheet microscopy (Ahrens et al., 2013), which would allow the study of how concerted activity patterns across large populations of neurons across the brain relate to behavior. In combination with analysis methods for high-dimensional data (Yu et al., 2009; Paninski et al., 2010), this would promise insights into how large neuronal populations process sensory information and generate behavior.

Zebrafish exhibit a variety of visually-driven behaviors, including innate behaviors such as the optomotor response (Orger et al., 2000, 2008), prey-capture (Bianco et al., 2011), and learned behaviors such as motor adaptation (Portugues and Engert, 2011; Ahrens et al., 2012) and associative learning (Aizenberg and Schuman, 2011). Discovery of novel behaviors is still ongoing. The virtual reality paradigm presented here makes it possible to transfer many behavioral assays to a microscope, allowing for whole-brain functional imaging and optogenetic perturbation of neural activity during naturalistic behavior.

## Methods

Larval zebrafish of 6–7 days post fertilization were paralyzed by immersion in a drop of E3 solution with 1 mg/ml alpha-bungarotoxin (Sigma-Aldrich) and embedded in a drop of 2% low melting point agarose in the lid of a 30 mm dish or on a 60 mm acrylic surface (Video S2), after which the tail was freed by cutting away the agarose around it. Alternatively, they were suspended from three structural pipettes. Four electrodes (two in the case of suspension from structural pipettes)—suction pipettes of diameter 45 micrometers—were placed on the tail of the fish at intersegmental boundaries, and gentle suction applied until electrical contact with the motor neurons axons was made, usually after about 10 min. These electrodes allowed for the recording of multi-unit extracellular signals from clusters of motor neuron axons, and provided a potential readout of intended locomotion (Masino and Fetcho, 2005; Ahrens et al., 2012). Underneath the petri dish containing the fish was a diffusive screen onto which visual stimuli could be projected via a mini projector, similar to the setup reported before (Ahrens et al., 2012).

Extracellular signals were amplified with a Molecular Devices Axon Multiclamp 700B amplifier and fed into a computer using a National Instruments data acquisition card. Custom software written in C# (Microsoft) processed the incoming signals in real time, and updated the visual stimulus using DirectX. Electrophysiological signals, as well as stimulus and virtual fish parameters (x and y position, angle, stimulus type) were written to the hard disk. Fictive swim bouts were processed as described previously (Ahrens et al., 2012), separately for the left and the right channels. When recording with four channels, the channel with the best signal to noise ratio on each side of the fish was selected. The visual stimulus was updated at 60 frames/s, and incoming electric signals were processed at the same rate, in chunks of about 17 ms. For each chunk of data, the local power (in a 10 ms sliding window) was computed and thresholded, by an automatic threshold computed as described previously (Ahrens et al., 2012). For any signals passing the threshold, the weighted difference in power between the left and the right channels determined the change in virtual heading angle, Δangle = *g*_left_ × *P*_left_ − *g*_right_ × *P*_right_. The sum of the power of both channels determined the displacement in the forward direction, *g*_forward_ × (*P*_left_ + *P*_right_). The gain parameters *g*_left_, *g*_right_, and *g*_forward_ were set so that (1) the first response to a forward grating would displace the fish about 1 cm forward [approximately matched to free swimming behavior; the fish was about 1.2 cm above the display (which also determines the perceived optic flow)], (2) the first response to a left (right) grating would cause a turn angle of 35° (approximately matched to free swimming behavior). This calibration was repeated approximately every 20 min, depending on the stability of the recordings. An upper bound to the angle change of 20° per 17 ms was applied to avoid very large angle turns. Visual stimuli used were of three types. Gratings consisted of red and black bars 1 cm wide. The stimulus for the 2D OMR is shown in Video S2. The stimulus comprising the light-modulated environment is shown in Figures 6, 7.

Two-photon imaging was performed using a custom built two-photon microscope. For imaging, transgenic zebrafish, in the *nacre* background, expressing GCaMP5G (Akerboom et al., 2012) under the *elavl1* [formerly known as HuC (Park et al., 2000)] promoter were used. To avoid interference of the visual stimulus and the photon detection, the photomultiplier tube of the microscope was protected by a green-pass optical filter. In addition, the light source of the mini projector delivering visual stimulation was replaced by a red light emitting diode (LED), which was pulsed at the same frequency as the fast scan mirror, to deliver a light pulse when the laser beam was directed to the left edge of the imaged plane (typically at 800 Hz). Two-photon image acquisition occurred via software, custom written in C#, and was synchronized to the electrophysiology recording and stimulus delivery via digital pulses.

## Author contributions

Misha B. Ahrens, Kuo-Hua Huang, and Florian Engert conceived the experiments. Misha B. Ahrens and Kuo-Hua Huang performed the experiments and analyzed the data. Misha B. Ahrens and Florian Engert wrote the manuscript with help of Kuo-Hua Huang, Brett D. Mensh, and Sujatha Narayan.

### Conflict of interest statement

The authors declare that the research was conducted in the absence of any commercial or financial relationships that could be construed as a potential conflict of interest.
